# Light Exposure Ameliorates Tau-Induced Deficits via Adenosine Signaling and Mitochondrial Quality Control in *Drosophila*

**DOI:** 10.3390/biomedicines14071502

**Published:** 2026-07-02

**Authors:** Su Zhang, Yuanhang Xiang, Xinxin Huang, Chuncao Ao, Linfeng Chen, Xinhui Zhang, Zhong Li

**Affiliations:** 1 Department of Neurology, The Sixth Affiliated Hospital, Sun Yat-sen University, Guangzhou 510655, China; 2 Sino-French Hoffmann Institute, School of Basic Medical Science, Guangzhou Medical University, Guangzhou 511436, China; 3 Department of General Surgery, First Affiliated Hospital of Gannan Medical University, Ganzhou 341000, China; 4 Key Laboratory of Human Microbiome and Chronic Diseases, Sun Yat-sen University, Ministry of Education, Guangzhou 510655, China; 5 Biomedical Innovation Center, The Sixth Affiliated Hospital, Sun Yat-sen University, Guangzhou 510655, China; 6 Guangdong Provincial Key Laboratory of Brain Function and Disease, Guangzhou 510080, China; 7 Shenzhen Research Institute, Sun Yat-sen University, Shenzhen 518000, China

**Keywords:** light exposure, adenosine signaling, mitochondrial quality control, tau, *Drosophila melanogaster*

## Abstract

**Background**: Accumulating evidence suggests that environmental light cues influence brain function and neurodegenerative processes; however, the underlying cellular mechanisms remain incompletely understood. **Methods**: Here, using a Tau-overexpressing *Drosophila* model, we investigated how light exposure modulates neurodegeneration-associated phenotypes, with a particular focus on adenosine signaling and mitochondrial homeostasis. We performed behavioral assays, biochemical measurements, genetic interference targeting the adenosine receptor, and mito-QC reporter analysis to assess mitochondrial quality control. **Results**: We show that light exposure ameliorates Tau-induced behavioral impairments and neuropathological features, reducing climbing time by approximately 29% in males and 45% in females, and extending median lifespan by ~29% in males and ~26% in females. Notably, biochemical analyses revealed that light exposure significantly increases brain adenosine levels at ZT12 by approximately 5 to 6 nmol/L in both sexes (*p* < 0.01), suggesting a light-dependent modulation of adenosine availability. To further examine the role of adenosine signaling, we performed genetic interference experiments targeting the adenosine receptor. These results indicate that adenosine receptor-associated signaling is functionally involved in the beneficial effects of light, as disruption of this pathway attenuates the light-induced improvements in behavioral and mitochondrial phenotypes. Using a mito-QC reporter system, we further show that light exposure enhances mitochondrial quality control, as reflected by a ~2.3-fold increase in mitolysosome density (*p* < 0.001). Importantly, this effect is modulated by the functional state of adenosine signaling, suggesting a potential interaction between these processes. **Conclusions**: Together, our findings indicate that light exposure is associated with coordinated changes in adenosine signaling and mitochondrial quality control, which may contribute to the attenuation of Tau-induced deficits in *Drosophila*. This work provides insight into how environmental light cues may influence neurodegeneration-related cellular processes and highlights the potential relevance of light-based interventions for future mechanistic and translational studies.

## 1. Introduction

Neurodegenerative disorders are characterized by progressive neuronal dysfunction and loss, often accompanied by impairments in cellular energy metabolism and mitochondrial homeostasis [1,2]. Among these diseases, tauopathies represent a major class of neurodegenerative conditions in which abnormal accumulation of Tau protein disrupts neuronal integrity, synaptic function, and intracellular trafficking [3,4]. Mitochondrial dysfunction is increasingly recognized as a prominent contributor to neurodegeneration-associated neurotoxicity, including tau-related pathology, through altered bioenergetics, oxidative stress, and defective mitochondrial quality control [1,2]. Mitochondrial quality control, including mitochondrial dynamics and selective removal of damaged mitochondria through mitophagy, is essential for maintaining neuronal homeostasis. Dysregulation of mitophagy has been implicated in multiple neurodegenerative conditions and is thought to exacerbate tau-associated pathology by permitting the accumulation of dysfunctional mitochondria [5,6,7]. Despite its importance, the upstream modulators that influence mitophagy and mitochondrial homeostasis in tauopathy remain incompletely understood.

Environmental factors have emerged as important modulators of brain physiology and neurodegenerative processes. Light exposure, in particular, is a potent regulator of circadian rhythms, neuronal activity, and metabolic states. Beyond its role in circadian entrainment, accumulating studies suggest that light can influence mitochondrial function, oxidative metabolism, and neurobehavioral outcomes in both animal models and humans [8,9]. However, the cellular and molecular mechanisms by which light exposure affects mitochondrial homeostasis and neurodegeneration-associated phenotypes remain largely undefined.

Adenosine (Ado) is a ubiquitous neuromodulator that integrates metabolic state, neuronal activity, and cellular stress responses. In the nervous system, adenosine signaling plays critical roles in regulating synaptic transmission, energy balance, and neuroprotection [10,11]. Alterations in adenosine homeostasis have been reported in aging and neurodegenerative conditions, and adenosine receptor-associated signaling has been implicated in the regulation of mitochondrial function and autophagic processes [12,13,14]. The *Drosophila melanogaster* model, with its single adenosine receptor and circadian rhythm analogous to humans, provides an excellent genetic system for analyzing these mechanisms. Manipulating AdoR in flies significantly alters sleep patterns and neuronal resilience [15,16]. Notably, beyond its role in regulating neuronal excitability, adenosine signaling is crucial for cellular energy homeostasis and mitochondrial function [17,18]. The emergence of adenosine as a key element in the pathogenesis of neurodegenerative diseases has been demonstrated to be strongly linked to mitochondrial dysfunction, which is characterized by impaired energy production, increased oxidative stress, and defective quality control mechanisms [1,2]. A central mechanism in this control is mitophagy, the selective autophagy of damaged mitochondria governed by the PINK1/Parkin pathway [19,20,21,22]. These observations raise the possibility that adenosine signaling may serve as a functional link between environmental cues, such as light exposure, and mitochondrial quality control.

In this study, we use a Tau-overexpressing *Drosophila* model to investigate how light exposure influences Tau-induced deficits and mitochondrial homeostasis. By combining behavioral analyses, biochemical measurements, genetic interference approaches, and in vivo mitophagy reporters, we explore the functional relationship between light-dependent modulation of adenosine-associated signaling and mitochondrial quality control. Our findings provide insight into how environmental light cues may shape neurodegeneration-related cellular processes and highlight mitochondrial homeostasis as a potential target of light-dependent modulation.

## 2. Materials and Methods

### 2.1. Fly Stocks and Husbandry

The fly strains *nSyb-Gal4*, *UAS-Tau^R406W^*, and *UAS-APP*, *BACE1* were kindly provided by Dr. Yuanhang Xiang (Sino-French Hoffmann Institute, Guangzhou Medical University, Guangzhou, China). The *UAS-mito-QC* strain was provided by Dr. Chuanxian Wei (Sino-French Hoffmann Institute, Guangzhou Medical University, Guangzhou, China). The *elav-Gal4 > UAS-Aβ_42_ H29.3* strain was provided by Dr. Yi Zhong (Tsinghua University, Beijing, China). The *UAS-mCherry-RNAi* (BDSC #35785), *UAS-AdoR-RNAi* (BDSC #27536) and *Pink1^B9^* (BDSC #34749) lines were obtained from the Bloomington *Drosophila* Stock Center (BDSC); the latter has been previously validated for *AdoR* knockdown efficacy [15]. All transgenic lines were maintained in a *w^1118^* genetic background.

*Drosophila* melanogaster fly stocks were maintained using standard procedures in a temperature- and humidity-controlled incubator under a 12 h:12 h light/dark cycle at 25 °C. Flies were raised on standard cornmeal-based media. For adenosine supplementation experiments, the final concentration of adenosine in the fly food was 1.65 mM (500 µg/mL), as previously described [23]. Briefly, adenosine was dissolved in water and mixed into the fly food. All experimental crosses were conducted at 25 °C, and both male and female flies were used in the experiments. All flies were collected under ice-induced anesthesia and sorted into vials at a density of 20 flies per vial by sex. Flies were transferred to fresh food every 2 days.

Different light treatments were carried out during the 12 h light period (from zeitgeber time [ZT] 0 to ZT 12 each day). A 10-day photic intervention was administered to 20-day-old flies. For bright light treatment (BLT), flies were exposed to white light at 1000 lux throughout the light phase only (ZT0-ZT12; BLT+ group) using LED light panels (correlated color temperature: 4000–5000 K), while controls without BLT were maintained under standard incubator illumination at approximately 50 lux during the same light phase (BLT− group). The intensity of 1000 lux was selected based on preliminary dose–response experiments testing 200, 500, 1000, and 2000 lux, in which 1000 lux provided the most robust improvement in climbing performance and survival without phototoxicity. The dark phase (ZT12-ZT24) was kept dark for all groups. Temperature was monitored throughout the experiment to ensure no thermal effects from the light source (maintained at 25 ± 0.5 °C).

### 2.2. Lifespan and Climbing Activity Assay

For lifespan assays, newly eclosed flies were collected and separated by sex. Male and female flies were divided into groups of 20 per vial. These flies were maintained on standard food at 25 °C and transferred to fresh vials every 2 days. Survival was monitored daily, and lifespan curves were analyzed using Kaplan–Meier curves in GraphPad Prism 10.1.2. Statistical significance between groups was determined using the log-rank (Mantel–Cox) test.

For climbing activity assay, 20 male or female adult flies from each treatment group were placed in glass cylinders (2.5 cm diameter, 25 cm height). Flies were transferred to the testing tubes using gentle aspiration without anesthesia to avoid potential effects of anesthetics on locomotor performance. Prior to testing, flies were allowed to acclimate to the testing apparatus for approximately 2 min to minimize handling-induced stress. All experimental groups were subjected to the same handling and acclimatization procedures to ensure consistency across experiments. Following the acclimation period, flies were gently tapped to the bottom of the cylinder, and the time (in seconds) taken by the flies to climb vertically for 12 cm was recorded. This procedure was repeated for nine consecutive trials with consistent inter-trial intervals. For statistical analysis, measurements were summarized per biological replicate (vial), as described in the figure legends.

### 2.3. Behavioral Experiments and Analysis

Aversive olfactory conditioning was performed in a T-maze according to a Pavlovian protocol as previously described [24]. Behavioral experiments were performed during the daytime within a fixed time window (ZT3–ZT7) under controlled light conditions. By maintaining a consistent testing period for all experimental groups, potential circadian influences were minimized. Groups of 50–60 flies (30 days old) were placed in a training tube equipped with an electrified copper grid. During training, flies were exposed for 60 s to 0.1% 3-octanol in mineral oil, as the conditioned stimulus (CS^+^), paired with twelve pulses of 90 V electric shock (each 1.25 s duration, with a 3.75 s inter-pulse interval). This was followed by a 30 s rest period and then 60 s exposure to 0.15% 4-methylcyclohexanol in mineral oil as the control conditioned stimulus (CS^−^) without any shock. A single cycle of this procedure was sufficient to induce short-term memory, which was tested immediately afterward under dim red light at 70% humidity. Flies were allowed to choose between the two odor arms for 120 s. A performance index (*PI*) is calculated as:

$$PI=\frac{\left(\mathrm{number}\;\mathrm{of}\;CS^{-}\;\mathrm{flies}-\mathrm{number}\;\mathrm{of}\;CS^{+}\;\mathrm{flies}\right)}{\mathrm{total}\;\mathrm{number}\;\mathrm{of}\;\mathrm{flies}\;\mathrm{in}\;\mathrm{both}\;\mathrm{arms}}$$

*PI* of 0 indicates no odor preference, while a *PI* of 1 represents complete avoidance of CS^+^ odor. Baseline assays were carried out on naive flies with approximately 50 individuals per trial under identical conditions. These included an odor preference test, wherein flies chose between 3-octanol (OCT) or 4-methylcyclohexanol (MCH) and clean air, scored as the percentage choosing odor minus the percentage choosing air; and a shock sensitivity test, in which flies chose between a shock-paired tube delivering 90 V pulses (1.25 s pulses every 5 s) and a safe tube over 2 min, scored as the percentage avoiding shock minus the percentage choosing the safe tube.

### 2.4. Immunostaining and Image Analysis

The measurement of brain neurodegenerative vacuoles was performed based on protocols described in a previous study [25]. Briefly, whole flies were fixed in 4% paraformaldehyde with 0.5% Triton X-100 for 3 h, washed thoroughly, and dissected in 0.008% Triton X-100 buffer. Isolated brains were incubated overnight at 4 °C with a staining solution containing Phalloidin (1:100, 12877s, CST, Danvers, MA, USA) and DAPI (1:1000, C1002, Beyotime, Shanghai, China) to label the actin-rich neuropil and nuclei, respectively. After washing, brains were mounted anterior side up using an antifade mountant on imaging spacers and imaged via confocal microscopy (1024 × 1024, 1 µm Z-step size) to obtain full Z-stacks of the entire brain. Vacuoles were identified as regions within the neuropil that were negative for both phalloidin and DAPI. ImageJ was used to trace and quantify vacuole area and number in the largest cross-sectional plane per vacuole.

To measure mitochondrial content (ATP5a) and mitophagy (mito-QC) in *Drosophila* brains, the brains were fixed in 3% formaldehyde in PBS for 20 min. The brains were then dissected and post-fixed for 5 min. They were permeabilized and blocked in PBST with 5% NGS and incubated overnight at 4 °C with mouse anti-ATP5a (1:250, 15H4C4, abcam) to label the mitochondria. After washing in PBST, the samples were incubated with DAPI (1:1000) for 45 min at room temperature. The samples were then mounted in Vectashield and imaged using a Zeiss LSM880 confocal microscope (Oberkochen, Germany). ImageJ was used to quantify fluorescence intensity and mitochondrial area from confocal stacks acquired under identical settings.

For mitophagy, flies carrying the outer-membrane-targeted GFP-mCherry (mito-QC) reporter [26] were dissected in cold Schneider’s medium. The brains were then mounted in the same medium and imaged using a Zeiss LSM880 confocal microscope. Mitolysosomes were scored as mCherry-only puncta (where GFP is quenched in the acidic lysosome) using ImageJ and were typically normalized to a defined brain area.

### 2.5. Adenosine-Level Measurement

Adenosine concentrations in the heads of *Drosophila* were measured 10 days after light intervention. Adult flies aged 30 days were briefly anesthetized with CO_2_ and decapitated on ice. For each biological replicate, 50 heads were collected, with six replicates performed. To minimize post-mortem adenosine release from ATP degradation, heads were immediately homogenized on ice in ice-cold PBS containing protease and phosphatase inhibitors. The lysates were then centrifuged at 12,000× *g* for 10 min at 4 °C, and the resulting supernatant was collected. Commercial ELISA kits (Meibiao Biology, Yancheng, China) were used according to the manufacturer’s instructions to measure adenosine concentrations in the fly heads. Absorbance was measured at the recommended wavelength using a microplate reader, and adenosine concentrations were calculated based on a standard curve generated from known adenosine standards. While ELISA-based measurement has inherent limitations, it provides a reliable relative comparison between treatment groups processed under identical conditions.

### 2.6. Western Blot

Adult flies aged 30 days were briefly anaesthetized with CO_2_ and decapitated using liquid nitrogen. For each biological replicate, 50 heads were collected, with at least three replicates performed. The heads were then homogenized on ice in ice-cold RIPA buffer containing protease and phosphatase inhibitors. The lysates were then centrifuged at 12,000× *g* for 10 min at 4 °C and the resulting supernatant was collected for protein quantification using a BCA assay. Equal amounts of protein were denatured in Laemmli buffer at 95 °C for 5 min. The proteins were separated by SDS-PAGE. The gels were then transferred to methanol-activated PVDF membranes at 100 V for 60–90 min at 4 °C. The membranes were then blocked with 5% BSA or skim milk in TBST for 1 h at room temperature. Primary antibodies were incubated overnight at 4 °C, followed by HRP-conjugated secondary antibodies for 1 h at room temperature.

Antibody-antigen binding was assessed by an enhanced chemiluminescence Image Quant LAS 4000 imager (GE Healthcare Bio-Sciences, Sweden), and the results were quantitively analyzed by ImageJ 1.53t software. The following primary antibodies were used (Table 1):

The secondary antibody was HRP-conjugated goat anti-rabbit antibody (1:5000, 31,460, Thermo Scientific, Waltham, MA, USA) or HRP-conjugated goat anti-mouse antibody (1:5000, 31,430, Thermo Scientific, Waltham, MA, USA). The levels of the target proteins were normalized to those of *β*-actin.

### 2.7. Transmission Electron Microscopy

The brains of *Drosophila* were dissected in ice-cold PBS and fixed immediately in 2.5% glutaraldehyde in a 0.1 M phosphate buffer solution (pH 7.4) at 4 °C overnight. The samples were then washed three times in 0.1 M phosphate buffer and post-fixed with 1% osmium tetroxide in the same buffer at room temperature for 2 h. After fixation, the tissues were dehydrated through a graded ethanol series, with each step taking 10 min. Dehydration was completed with two changes of absolute acetone. The samples were infiltrated with a 1:1 mixture of epoxy resin and acetone for 4 h at 37 °C, followed by pure resin overnight. The next day, the tissues were embedded in fresh epoxy resin and polymerized at 60 °C for 48 h. Ultra-thin sections were cut using a diamond knife and collected onto copper grids. Prior to imaging, the sections were stained with uranyl acetate and lead citrate. Images were captured using a Hitachi HT7800/HT7700 transmission electron microscope (Hitachi High-Tech Corporation, Tokyo, Japan).

### 2.8. Statistics

Statistical analyses were performed using GraphPad Prism (v10.1.2, San Diego, CA, USA). The Shapiro–Wilk test was used to assess data normality. Data are presented as mean ± SD. Comparisons between two independent groups were performed using unpaired two-tailed Student’s *t*-tests. For comparisons involving more than two groups, one-way analysis of variance (ANOVA) followed by Tukey’s post hoc test was applied to control for multiple comparisons. Survival data were analyzed using the log-rank (Mantel–Cox) test. A *p* value < 0.05 was considered statistically significant. Exact *p* values and sample sizes are reported in the figure legends.

## 3. Results

### 3.1. Light Exposure Attenuates Tau-Induced Behavioral Impairments and Neuropathological Features in Drosophila

The fruit fly *Drosophila* melanogaster, which shares numerous disease-associated genes with humans and possesses a stable circadian rhythm, serves as a well-established model for studying light exposure effects. Previous research indicates that higher-intensity visible light more robustly shapes the phase and stability of circadian rhythms, justifying our investigation under controlled, intense white light conditions [27]. Building on this, we first established that BLT has no adverse effects on normal control flies (*nSyb-Gal4/+*), as confirmed by lifespan and climbing assays (Supplementary Figure S1A,B). We then systematically evaluated BLT’s impact using a well-characterized Tau-overexpressing fly model [4] (*nSyb-Gal4 > UAS-Tau^R406W^*), which exhibits progressive neurodegeneration and premature mortality, providing a robust platform for assessing the effects of BLT.

**Figure 1:**
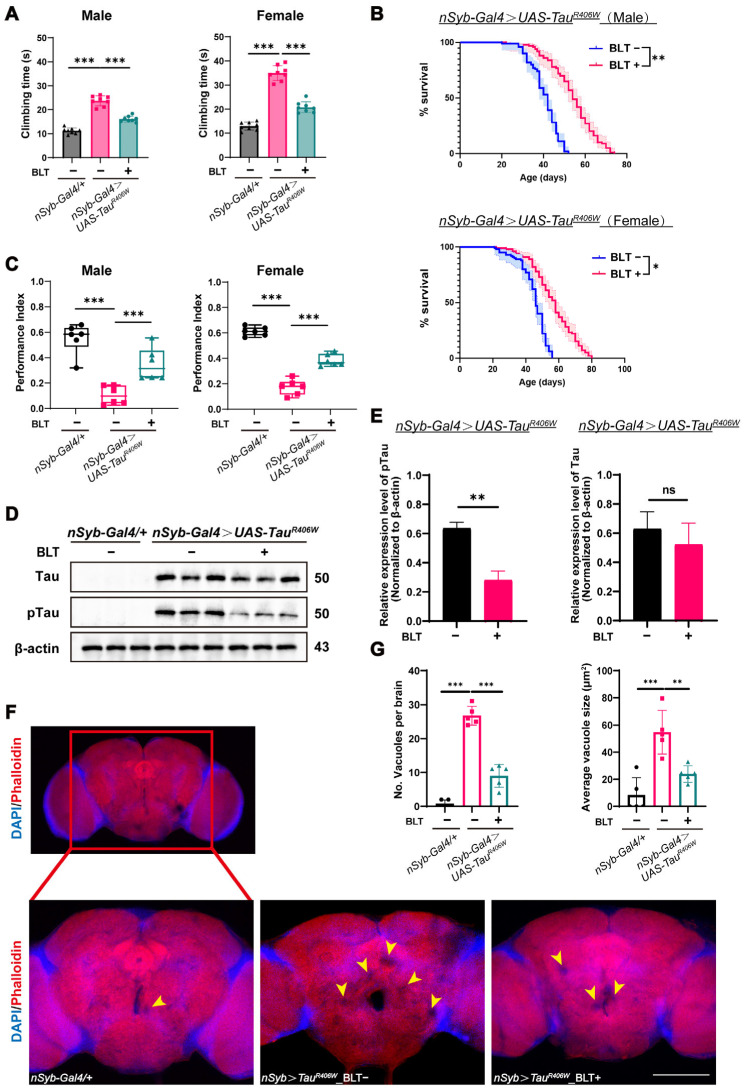
Light exposure ameliorates Tau-induced behavioral impairments and neuropathological features in *Drosophila*. (**A**) The negative geotaxis assay was used to assess locomotion behavior. BLT was found to significantly improve locomotor function, as demonstrated by reduced climbing time in both male and female flies. (**B**) Survival curves of male and female Tau-overexpressing flies (*nSyb-Gal4 > UAS-Tau^R406W^*) under bright light treatment (BLT+) or control condition (BLT−). BLT significantly prolonged the lifespan of both sexes. (**C**) Olfactory learning ability was evaluated using an odor avoidance test based on a T-maze. BLT-treated Tau-overexpressing flies exhibited a significantly higher performance index than their untreated counterparts, suggesting an improvement in olfactory-associated cognition. (**D**,**E**) Representative images of Western blots showing the expression of Tau and pTau protein. BLT reduces the levels of pTau expression in the heads of Tau-overexpressing flies. The expression levels of proteins were quantified. (**F**,**G**) Representative fluorescence microscopy images of the whole brain (**F**) and of representative vacuoles (yellow arrows). Scale bar, 100 µm. (**G**) BLT significantly reduced both the number and average size of vacuoles in the brains of Tau-overexpressing flies. * *p* < 0.05; ** *p* < 0.01; *** *p* < 0.001. ns, not significant. Data are presented as mean ± SD.

Our comprehensive assessment encompassed three key behavioral parameters: negative geotaxis/climbing ability, lifespan, and T-maze olfactory learning, along with histological assessment of phosphorylated Tau (pTau) protein and whole-brain neurodegenerative vacuoles. The results showed that BLT produced significant improvements across all measured parameters. BLT-treated flies demonstrated enhanced locomotor performance, with climbing time reduced by approximately 29% (from 22.99 ± 2.13 s to 16.39 ± 1.18 s, *p* < 0.001) in males and by approximately 45% (from 35.55 ± 2.65 s to 19.66 ± 1.34 s, *p* < 0.001) in females; extended lifespan, with median survival increased by approximately 29% (from 42 to 54 days, *p* < 0.01) in males and by approximately 26% (from 46 to 58 days, *p* < 0.05) in females; and improved T-maze performance, with performance index approximately doubled in both sexes (*p* < 0.001) (Figure 1A–C). We further validated the effects of BLT in multiple neurodegenerative disorder *Drosophila* models using lifespan and climbing experiments (Supplementary Figures S2A–C and S3A–C). Histological analysis revealed that BLT reduced pTau protein levels to about 56% of control levels (*p* < 0.01) and significantly decreased both the number and size of neurodegenerative vacuoles, with the vacuole count and area reduced by approximately 66% and 56% (Figure 1D–G). These concordant improvements at both behavioral and pathological levels demonstrate the capacity of BLT to alleviate Tau-induced phenotypes across multiple systems, establishing a solid foundation for subsequent mechanistic investigations.

### 3.2. Light Exposure Exerts Its Beneficial Effects in an AdoR-Dependent Manner

Having established BLT’s efficacy in attenuating Tau-induced phenotypes, we sought to elucidate its underlying molecular mechanism. Given the established role of adenosine as a key sleep- and arousal-related neuromodulator that accumulates during wakefulness, and its emerging importance in neurodegeneration-associated processes, we investigated whether adenosine signaling contributes to BLT-associated benefits. We therefore tested the hypothesis that BLT attenuates Tau-induced phenotypes, at least in part, through adenosine receptor (AdoR)-associated signaling. *Drosophila*, with a single, well-characterized *AdoR* gene, provides an ideal model for testing this hypothesis via genetic intervention [15].

**Figure 2:**
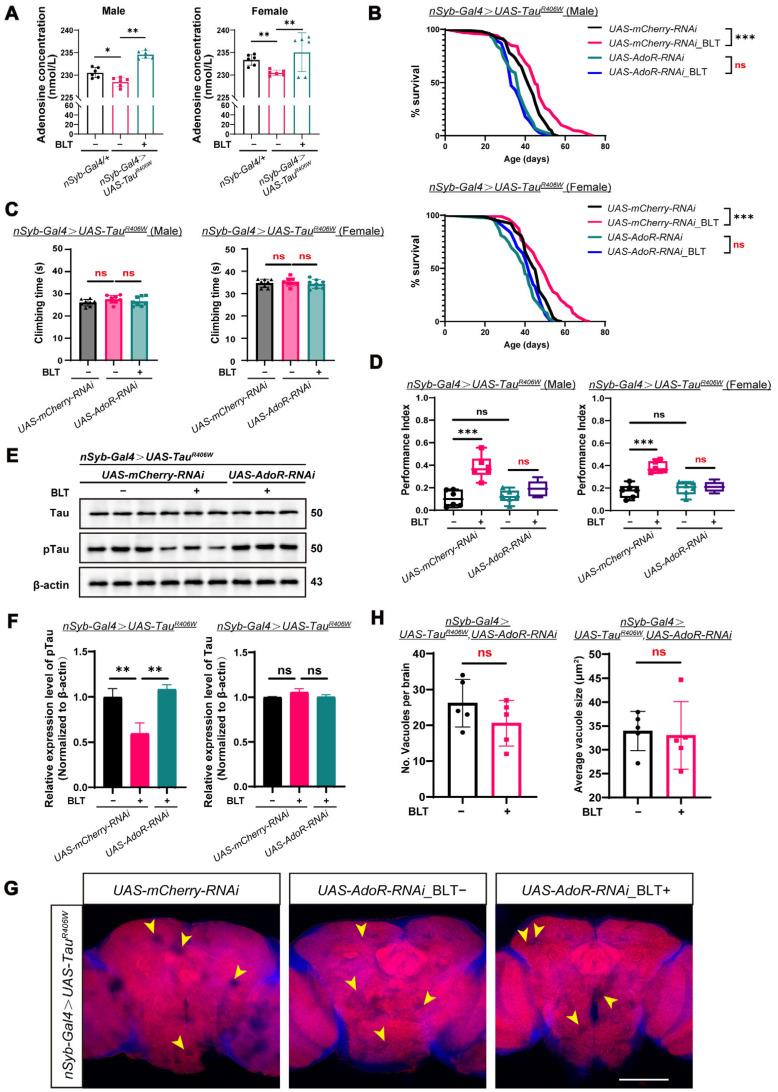
Light exposure exerts beneficial effects in an AdoR-dependent manner. (**A**) Quantification of head adenosine concentrations in male and female Tau-overexpressing flies under BLT− or BLT+ conditions at ZT12. BLT significantly increased adenosine levels in both sexes compared to untreated Tau-overexpressing flies. (**B**) Survival curves of male and female Tau-overexpressing flies co-expressing either *UAS-mCherry-RNAi* or *UAS-AdoR-RNAi* with or without BLT. Knockdown of *AdoR* negated the effect of BLT on lifespan. (**C**) Climbing ability assessed in male and female Tau-overexpressing flies with or without *AdoR* knockdown and with or without BLT treatment. (**D**) Olfactory memory performance in male and female Tau-overexpressing flies. BLT enhanced olfactory learning in *mCherry-RNAi* but not in *AdoR-RNAi* backgrounds, indicating the dependency of cognitive rescue on AdoR. (**E**,**F**) Representative images of Western blots showing the expression of Tau and pTau protein. BLT significantly reduced levels of pTau in *mCherry-RNAi* flies, but not in *AdoR-RNAi* flies. The expression levels of proteins were quantified. (**G**,**H**) Representative fluorescence microscopy images of whole-brain (**G**) and representative vacuoles (yellow arrows). Scale bar, 100 µm. (**H**) Following *AdoR* knockdown, the ameliorative effect of BLT on brain neurodegenerative vacuoles in the Tau-overexpressing flies was significantly diminished. * *p* < 0.05; ** *p* < 0.01; *** *p* < 0.001. ns, not significant. Data are presented as mean ± SD.

To determine whether adenosine mediates BLT effects and to evaluate its temporal dynamics, we quantified adenosine levels in fly heads at four time points across the light–dark cycle (ZT0, ZT6, ZT12, and ZT18). Time-course analysis revealed that BLT progressively increased adenosine during the light phase, with the most pronounced elevation at ZT12, while at ZT0 adenosine levels did not differ between BLT-treated and control flies, indicating that BLT enhances daytime accumulation rather than altering baseline levels. By ZT18 (6 h into the dark phase), adenosine levels in the BLT group had returned to near-baseline values, confirming the reversibility of the BLT-induced adenosine increase (Supplementary Figure S4). BLT significantly increased adenosine levels at ZT12 in both sexes compared to control light conditions (Figure 2A), providing biochemical evidence for altered adenosine availability following light exposure. Next, we tested the necessity of AdoR signaling using genetic epistasis. To confirm the efficacy of AdoR knockdown, we performed qRT-PCR on heads of 30-day-old flies of the relevant genotypes. *AdoR* mRNA levels were reduced by approximately 70% in *AdoR-RNAi* flies compared to *mCherry-RNAi* controls (Supplementary Figure S5A,B), confirming effective knockdown in our experimental system. In control Tau-overexpressing flies expressing *mCherry-RNAi*, BLT improved lifespan, climbing ability, and olfactory learning (Figure 2B–D). However, these benefits were largely abolished in Tau-overexpressing flies with *AdoR* knockdown (Figure 2B–D), consistent with AdoR-associated signaling being required for BLT-associated phenotypic improvements [15]. At the molecular and pathological levels, BLT reduced pTau levels and neurodegenerative vacuoles in control flies, but these effects were significantly attenuated in the *AdoR-RNAi* background (Figure 2E–H). Collectively, these data support a functional requirement for AdoR in multiple BLT-associated readouts, while recognizing that additional genetic validation (e.g., independent RNAi lines and rescue) will further strengthen specificity.

### 3.3. Light Exposure Restores Mitochondrial Homeostasis and Enhances Brain Mitophagy

As adenosine signaling is known to modulate cellular energy homeostasis and mitochondrial function, we next investigated whether this pathway regulates mitochondrial quality control. Thus, we hypothesized that AdoR activation might trigger PINK1/Parkin-mediated mitophagy, a primary pathway for eliminating damaged mitochondria that is frequently impaired in neurodegenerative conditions.

**Figure 3:**
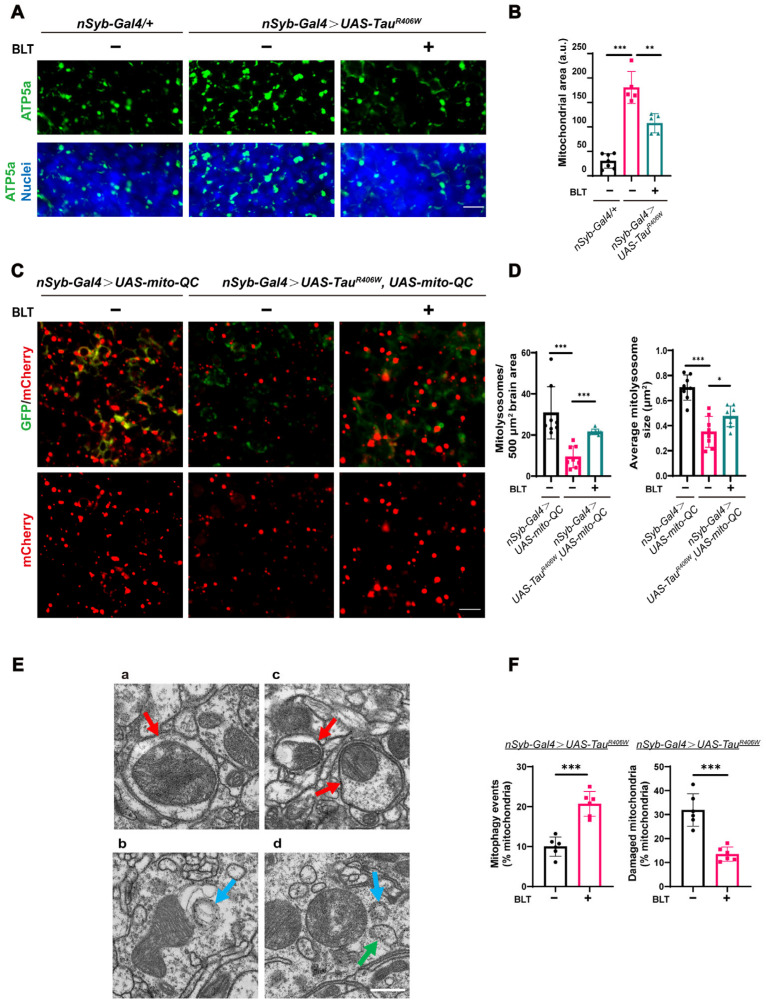
Light exposure improves mitochondrial homeostasis and is associated with increased mitophagy in the brains of Tau-overexpressing *Drosophila*. (**A**) Immunostaining of brains from control flies (*nSyb-Gal4/+*) and Tau-overexpressing flies with or without BLT, showing mitochondrial morphology (green channel, anti-ATP5a) and nuclear DNA (blue channel, stained with DAPI). Scale bar is 5 µm. (**B**) Quantification of mitochondrial area in brains as shown in (**A**). (**C**) Mito-QC imaging of brains from 30-day-old flies with or without BLT. Genotypes analyzed were *nSyb-Gal4 > UAS-mito-QC* (control) and *nSyb-Gal4 > UAS-Tau^R406W^*, *UAS-mito-QC*. Images show merged GFP (green) and mCherry (red) channels. Punctate mCherry-only foci (red, without green) indicate mitolysosomes, where GFP has been quenched in the acidic lysosomal environment. Scale bar, 5 µm. (**D**) Quantification of mitolysosome area per 500 µm^2^ and average size (µm^2^) as shown in (**C**). (**E**) Transmission electron microscopy (TEM) analysis of brains shows impaired mitophagy in Tau-overexpressing flies without BLT (blue arrow in (**b**)), increased mitophagic events in BLT-treated Tau-overexpressing flies (red arrows in (**c**)), as well as a potential phagophore (green arrow, (**d**)) engulfing damaged mitochondria (blue arrow, (**d**)). Mitophagy is also observed in control flies (red arrow, (**a**)). Scale bars: 500 nm. (**F**) The percentage of mitochondria undergoing mitophagy-like events and the percentage of damaged mitochondria. * *p* < 0.05; ** *p* < 0.01; *** *p* < 0.001. Data are presented as mean ± SD.

To assess mitochondrial structural integrity, we first performed immunofluorescence imaging using the ATP5a marker. BLT was associated with a shift in the mitochondrial network in Tau-overexpressing fly brains from a fragmented state to a more continuous architecture (Figure 3A). Quantitative analysis showed a significant reduction in ATP5a-defined mitochondrial area by approximately 40% (from 180.65 ± 32.84 to 107.64 ± 19.74, *p* < 0.01) (Figure 3B), consistent with reduced accumulation of enlarged and morphologically abnormal mitochondria.

This shift towards “normalized” morphology generally indicates balanced mitochondrial quality control, including fission–fusion coupling and selective clearance (i.e., mitophagy). Previous studies have revealed that enhanced mitophagy restores mitochondrial homeostasis by reversing age-related mitochondrial morphological and functional abnormalities in fly brains [28]. We then examined whether this structural improvement correlated with enhanced mitochondrial clearance using the mito-QC reporter system, an outer mitochondrial membrane-localized GFP-mCherry tandem probe. Since GFP is quenched in the acidic environment of lysosomes whereas mCherry remains fluorescent, this pH-sensitive probe enables specific tracking of mitolysosome formation through the appearance of mCherry-only puncta [29,30,31]. The results showed that BLT significantly increased both the number and area of these puncta in aged (30-day-old) Tau-overexpressing flies, with mitolysosome density increasing from 9.32 ± 5.28 to 21.53 ± 1.30 per 500 µm^2^ (~2.3-fold, *p* < 0.001), and average size increasing from 0.35 ± 0.12 µm^2^ to 0.47 ± 0.08 µm^2^ (~34% increase, *p* < 0.05) (Figure 3C,D), indicating enhanced mitophagic flux and mitochondrial turnover.

Transmission electron microscopy provided ultrastructural validation of these findings. BLT-treated Tau-overexpressing fly brains showed increased occurrences of mitochondria within autophagic structures, alongside a marked reduction in damaged mitochondria exhibiting characteristic features such as dense matrix, disorganized cristae, and abnormal swelling, with mitophagic events increasing from 9.99 ± 2.40% to 20.69 ± 3.10% of mitochondria (~107% increase, *p* < 0.001), while damaged mitochondria decreased from 31.91 ± 6.81% to 13.48 ± 2.96% (~58% reduction, *p* < 0.001) (Figure 3E,F). These morphological changes align with the established model that mitochondrial fragmentation can facilitate selective clearance [32,33]. To further assess whether these changes in mitophagy temporally correlate with the rise and fall of adenosine across the light–dark cycle, we extended the TEM quantification to ZT0 (adenosine trough) and ZT18 (post-light clearance). At ZT0, mitophagic events were minimal and comparable between BLT-treated and control flies. At ZT18, the BLT-induced mitophagic flux had largely subsided, mirroring the decline in adenosine levels (Supplementary Figure S6A,B). This close temporal correspondence between adenosine accumulation and mitophagic flux is consistent with the notion that BLT enhances mitochondrial quality control through adenosine-associated signaling. Together, these results indicate that BLT is associated with mitochondrial network reorganization and increased mitophagy-related readouts, supporting a link between BLT-associated phenotypic improvements and organelle-level homeostasis in Tau-induced pathogenesis.

### 3.4. Light Exposure Is Associated with PINK1/Parkin-Related Mitophagy Markers

The PINK1/Parkin pathway represents a major mechanism for targeted mitochondrial clearance [33]. Under conditions of mitochondrial damage, PINK1 stabilizes on the outer mitochondrial membrane and recruits Parkin, which ubiquitinates mitochondrial proteins to tag damaged organelles for autophagic degradation [33,34,35,36]. To investigate whether BLT enhanced mitophagy through PINK1/Parkin pathway, we assessed the expression of PINK1 and Parkin.

**Figure 4:**
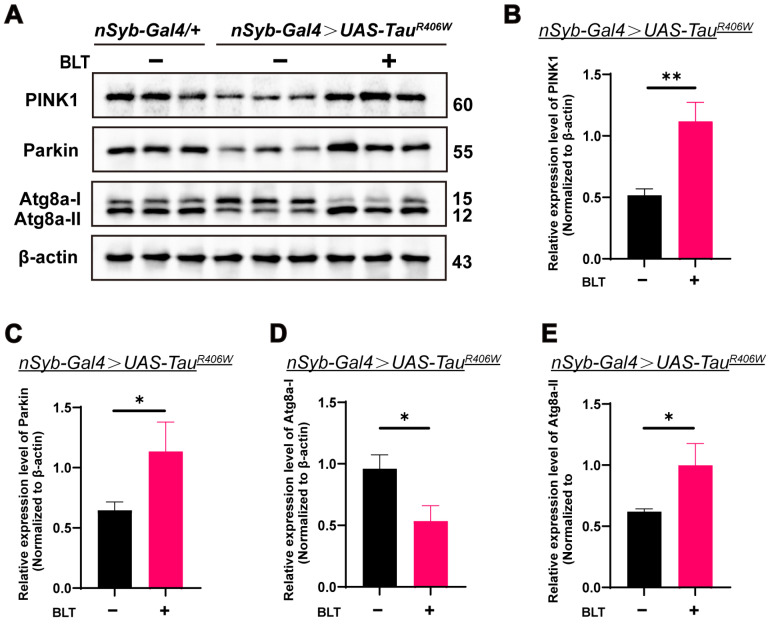
Light exposure is associated with PINK1/Parkin-related mitochondrial quality control and mitophagy in the brains of Tau-overexpressing *Drosophila*. (**A**) Representative images of Western blots showing the expression of PINK1, Parkin, Atg8a-I, and Atg8a-II protein. (**B–E**) Quantitative analysis of the levels of PINK1, Parkin, Atg8a-I, and Atg8a-II protein. * *p* < 0.05; ** *p* < 0.01. Data are shown as the mean ± SD.

Western blot analysis revealed that BLT significantly increased the protein levels of both PINK1 and Parkin in Tau-overexpressing fly heads, accompanied by an elevated Atg8a-II/Atg8a-I ratio (Figure 4A–E), a commonly used indicator of increased autophagosome formation. The coordinated upregulation of PINK1, Parkin, and Atg8a lipidation is consistent with enhanced engagement of mitochondrial quality control pathways [33,34,35,36].

### 3.5. PINK1 Is Required for BLT-Induced Mitophagy and Behavioral Rescue

**Figure 5:**
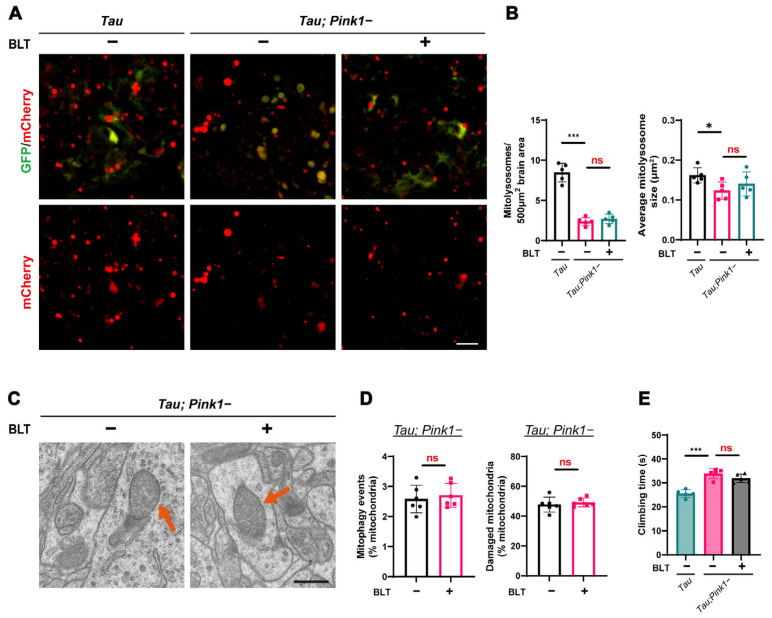
PINK1 is required for BLT-induced mitophagy and behavioral rescue. (**A**) Mito-QC imaging of brains from 30-day-old flies with or without BLT. *Tau* denotes *nSyb-Gal4 > UAS-Tau^R406W^*; *Pink1*− denotes *Pink1^B9^/Y* (male flies). Images show merged GFP and mCherry channels along with punctate mCherry-only foci. Scale bar, 5 µm. (**B**) Quantification of mitolysosome area per 500 µm^2^ and average size (µm^2^) as shown in (**A**). (**C**) TEM analysis of brains shows mitophagy in *Tau; Pink1*− flies under BLT− or BLT+ conditions. Orange arrows indicate damaged mitochondria. Scale bar, 500 nm. (**D**) The percentage of mitochondria undergoing mitophagy-like events and the percentage of damaged mitochondria. (**E**) Climbing ability assessed in male flies with or without BLT treatment. * *p* < 0.05; *** *p* < 0.001. ns, not significant. Data are presented as mean ± SD.

To determine whether PINK1 is functionally required for BLT-induced mitochondrial clearance and behavioral improvement, we generated Tau-overexpressing flies carrying the *Pink1^B9^* null allele and assessed mitophagy by mito-QC and TEM, as well as climbing performance, with or without BLT. In *Tau; Pink1*− mutants, BLT completely failed to induce mitophagy, with mitolysosome density and mitophagic events remaining at baseline regardless of BLT treatment (*p* > 0.05 for both) (Figure 5A–D).

The present study comprehensively examined the impact of BLT treatment on climbing performance in *Tau; Pink1*− mutants. The results revealed that the treatment did not result in any significant improvement in climbing times (33.84 ± 2.13 s vs. 31.89 ± 2.51 s, *p* > 0.05) (Figure 5E). It was observed that the baseline climbing ability of *Tau; Pink1*− flies was significantly worse than that of *Tau* flies (33.84 ± 2.13 s vs. 25.50 ± 1.18s, *p* < 0.001), suggesting that PINK1 loss exacerbates Tau-induced locomotor deficits even in the absence of light stimulation. The collective analysis of these data indicates that PINK1 is indispensable for both mitochondrial clearance and the behavioral improvement induced by BLT.

### 3.6. AdoR Signaling Is Functionally Involved in BLT-Associated Mitochondrial Homeostasis and Mitophagy Readouts

**Figure 6:**
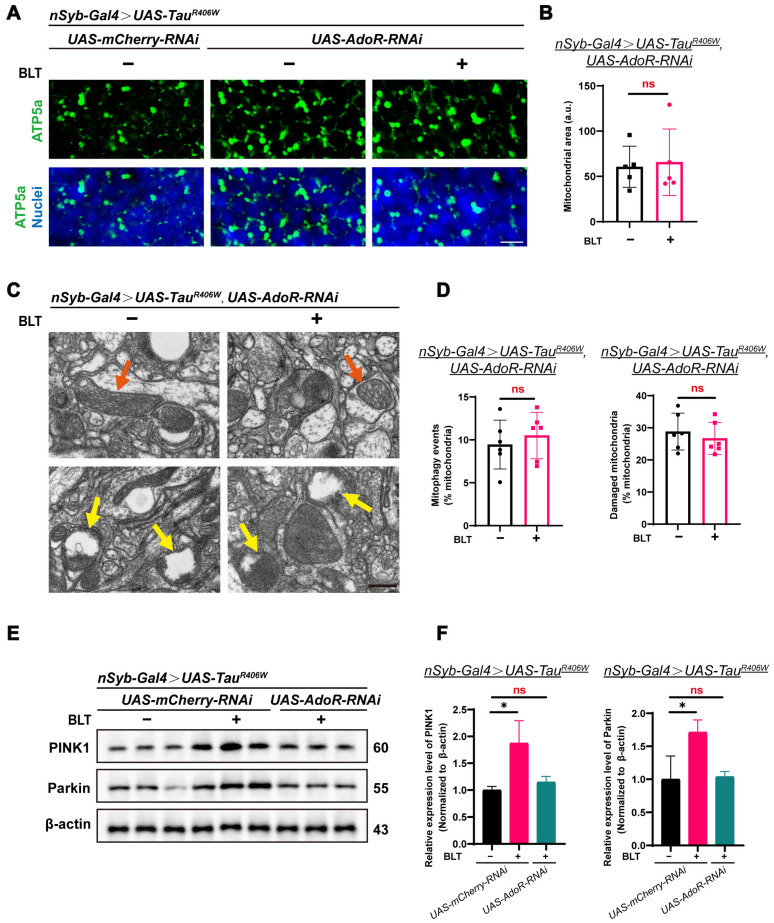
The beneficial effects of light exposure on mitophagy and mitochondrial homeostasis are functionally associated with adenosine signaling. (**A**) Immunostaining of brains from Tau-overexpressing flies with *mCherry-RNAi* or *AdoR-RNAi* under BLT− or BLT+ conditions, showing mitochondrial morphology (green channel, anti-ATP5a) and nuclear DNA (blue channel, stained with DAPI). Scale bar is 5 µm. (**B**) Quantification of mitochondrial area in brain as shown in (**A**). *AdoR* knockdown abolished the BLT-induced reduction in mitochondrial area. (**C**) TEM analysis of brains shows mitophagy in Tau-overexpressing flies with *AdoR-RNAi* under BLT− or BLT+ conditions. Orange arrows indicate moderately damaged mitochondria (slight dissolution and swelling but with an overall intact structure); yellow arrows indicate severely damaged mitochondria (severe dissolution, swelling, fragmentation, or complete loss of the inner membrane). Scale bar, 500 nm. (**D**) The percentage of mitochondria undergoing mitophagy-like events and the percentage of damaged mitochondria. (**E**) Representative images of Western blots showing the expression of PINK1 and Parkin protein. (**F**) Quantitative analysis of the levels of PINK1 and Parkin protein. * *p* < 0.05; ns, not significant. Data are shown as the mean ± SD.

We next investigated whether adenosine signaling contributes to BLT-associated effects on mitochondrial quality control. Genetic knockdown of *AdoR* abolished the BLT- associated improvement in mitochondrial morphology, as reflected by the persistence of enlarged mitochondrial area in AdoR-deficient flies (Figure 6A,B). Ultrastructural analysis further indicated that BLT did not reduce damaged mitochondria or increase mitophagy-like events in the absence of functional AdoR (Figure 6C,D). At the molecular level, *AdoR* knockdown attenuated BLT-associated upregulation of PINK1 and Parkin (Figure 6E,F).

To complement the necessity tests, we asked whether enhancing adenosine availability was sufficient to mimic BLT-associated benefits. To address this, we administered exogenous adenosine to Tau-overexpressing flies in the absence of BLT. Notably, adenosine supplementation alone partially recapitulated several BLT-associated phenotypes. Daytime administration of adenosine to aged Tau-overexpressing flies (from 20–30 days post-eclosion) significantly reduced neurodegenerative vacuoles and improved short-term memory (Supplementary Figure S7B–D). Consistent with these pathological improvements, mito-QC imaging revealed a marked increase in mCherry-only (mitolysosomes) puncta (Supplementary Figure S8A,B).

Taken together, these data support a functional involvement of AdoR-associated signaling in multiple BLT-associated outcomes, accompanied by changes in mitochondrial homeostasis and mitophagy-related readouts in a Tau-overexpressing *Drosophila* model. This signaling axis may represent a link between light-induced metabolic modulation and mitochondrial quality control.

## 4. Discussion

Alzheimer’s disease (AD), characterized by amyloid-*β* (A*β*) plaques, neurofibrillary tangles and significant neuronal loss, represents one of the most urgent public health challenges. Beyond these classical pathological features, sleep and circadian disturbances are increasingly recognized not only as early non-cognitive symptoms but also as potential accelerators of disease progression [37,38]. This dual role has prompted growing interest in non-pharmacological approaches, including bright light-based interventions, as potential modulators of disease-associated processes. Although clinical studies have reported that bright light can improve sleep and cognitive performance in individuals with AD [9,39,40], the underlying mechanisms, particularly at the molecular and organellar levels, remain incompletely defined.

In the present study, using a Tau-overexpressing *Drosophila* model, we investigated how light exposure influences neurodegeneration-associated phenotypes and mitochondrial homeostasis. Our data indicate that light exposure is associated with improved behavioral performance and attenuation of neuropathological features, accompanied by altered adenosine availability and enhanced mitochondrial quality control. Rather than defining a linear signaling pathway, our findings support a functional link between light-dependent metabolic modulation, adenosine receptor-associated signaling, and mitophagy-related processes, providing mechanistic insight into how light exposure may influence organelle-level pathology relevant to neurodegeneration.

Sleep and circadian disturbances are well-established targets of light-based interventions in clinical practice. A long-term randomized controlled trial demonstrated that increasing daytime light exposure to approximately 1000 lux slowed cognitive decline, improved neuropsychiatric symptoms, and enhanced sleep efficiency in older adults with dementia, with outcomes further optimized by low-dose melatonin supplementation [9]. Similarly, recent meta-analyses confirmed that light therapy improves sleep, cognitive function, and behavioral symptoms in AD patients [41]. From a physiological perspective, adenosine is a key regulator of sleep homeostasis, accumulating during prolonged wakefulness and modulating arousal networks via adenosine receptors [13,14]. In this context, our observation that light exposure increases head adenosine levels and is associated with improved tau-associated phenotypes in *Drosophila* suggests that modulation of adenosine availability may contribute to BLT-associated benefits. Together, these findings are consistent with a broader conceptual framework in which light-dependent regulation of metabolic and arousal-related signaling influences neuronal homeostasis, offering a mechanistic perspective for the benefits of light-based interventions.

From a mechanistic perspective, we demonstrated that light exposure is associated with enhanced mitochondrial quality control, concomitant with improvements in locomotor performance, lifespan, and memory in a Tau-overexpressing *Drosophila* model. A key conceptual advance of our study lies in identifying adenosine as a potential molecular bridge connecting light exposure to organelle clearance. AdoR is a G protein-coupled receptor that has been reported to stimulate adenylate cyclase and increase intracellular cAMP levels in *Drosophila*. In the present study, although we did not directly assess cAMP or PKA activity, our genetic data suggest that AdoR signaling is functionally involved in light-induced mitochondrial homeostasis and mitophagy. The precise downstream signaling mechanisms linking AdoR activation to mitochondrial quality control, including the potential involvement of cAMP/PKA-dependent or -independent pathways, remain to be elucidated in future studies.

Our genetic and pharmacological analyses support a functional involvement of AdoR signaling in the observed BLT-induced phenotypic improvements. Knockdown of *AdoR* abolished BLT-induced mitophagy, prevented a reduction in mitochondrial damage, and negated behavioral improvements. Conversely, exogenous adenosine administration partially recapitulated the beneficial effects of BLT, reducing neurodegenerative vacuoles, improving short-term memory, and enhancing mitophagic flux. These findings suggest that AdoR may function upstream of PINK1/Parkin activation, revealing how a systemic environmental cue (i.e., bright light) may converge on a cell-autonomous quality control pathway.

Our data support a model of induced, selective mitophagy, whereby mitochondria are targeted for lysosomal degradation following adenosine priming, rather than an increase in basal turnover. This distinction carries significant mechanistic and translational implications: while the PINK1/Parkin pathway is not essential for basal mitophagy in many tissues, it becomes crucial under stress conditions [26,35,42,43]. The observed BLT-induced increase in mitophagic activity coupled with reduced mitochondrial damage aligns with a quality-control response that supports mitochondrial fitness and synaptic resilience. The requirement of PINK1 for BLT-induced mitophagy and behavioral rescue further supports the hypothesis that the light–adenosine–mitophagy axis converges on the canonical PINK1-dependent mitochondrial quality control pathway. Notably, while the present data establish PINK1 as a critical downstream effector of AdoR signaling, the possibility that parallel pathways such as alternative mitophagy receptors (e.g., BNIP3/NIX) also contribute to the observed effects cannot be excluded. The residual mitophagy detected in *Tau; PINK-* mutants likely reflects PINK1-independent basal turnover, which is insensitive to BLT. Further studies using parkin mutants or double knockouts will provide further clarification of the hierarchical organisation of this pathway.

More broadly, our work supports a conceptual link between sleep- and metabolism- related signaling and organelle homeostasis. The combined application of mito-QC reporters, transmission electron microscopy, and autophagy flux analyses provided multilevel evidence that light exposure is associated with improved mitochondrial quality control. Rather than implying a direct disease-modifying effect, these findings suggest that light exposure may modulate endogenous cellular pathways that influence energy balance, oxidative stress, and Tau-associated toxicity. From this perspective, light-based interventions may have the potential to influence disease-relevant processes by acting at the level of organelle maintenance, rather than solely providing symptomatic relief.

These observations also have implications for future translational research. For example, exposure to moderate-intensity morning light (approximately 1000 lux), coupled with reduced evening blue-light exposure, aligns with human circadian biology and may support sustained modulation of adenosine-associated signaling. In addition, genetic variation in pathways related to adenosine metabolism or mitochondrial quality control could potentially influence individual responsiveness to light-based interventions, highlighting opportunities for biomarker-guided stratification. Combinatorial strategies integrating light exposure with other interventions known to influence mitophagy, such as pharmacological agents or physical activity, may warrant exploration in controlled, factorial study designs.

While our data support a functional involvement of AdoR signaling, we cannot exclude the contribution of other light-responsive pathways. For instance, light exposure may also influence mitochondrial function through direct photobiomodulation effects, modulation of circadian clock components (e.g., cryptochrome and timeless), or alterations in other neuromodulatory systems. Future studies employing cryptochrome mutants or tissue-specific manipulations will be needed to dissect these possibilities.

In addition, the effects of adenosine on mitochondrial quality control may vary depending on the biological context, including factors such as cell type, metabolic state, and receptor subtype involved. In mammalian systems, the presence of four adenosine receptor subtypes (A1, A2A, A2B, and A3) may contribute to such functional diversity. In contrast, *Drosophila* possesses a single AdoR, which simplifies receptor-specific signaling and provides a genetically tractable framework to examine adenosine-associated regulation of mitochondrial quality control. It is evident that, despite the single AdoR system in *Drosophila* providing a robust and genetically tractable platform for investigating the core adenosine–mitophagy signaling cascade, it is unable to fully replicate the complexity of mammalian adenosine receptor biology. Consequently, the findings should be interpreted as demonstrating the concept that adenosine-associated signaling can modulate mitochondrial quality control, rather than as a direct prediction of which mammalian receptor subtypes mediate analogous effects. Further studies employing subtype-selective pharmacological agents, receptor-specific knockout mice, or human neuronal models will be necessary to ascertain whether A1, A2A, A2B, A3, or combinatorial receptor activation underlies the light–adenosine–mitophagy axis in mammals.

Energy-sensing pathways may also participate in linking adenosine signaling to mitochondrial quality control. In particular, AMP-activated protein kinase (AMPK), a central regulator of cellular energy status, has been widely implicated in the control of autophagy and mitophagy. Although AMPK signaling was not directly examined in the present study, it remains a plausible intermediary through which changes in adenosine availability could influence mitochondrial turnover, and this possibility warrants further investigation. In particular, the elevation of adenosine triggered by BLT could, by signaling a shift in the energy charge of the cell, activate AMPK. This, in turn, may promote mitophagy through either PINK1/Parkin-dependent or alternative pathways. The temporal coincidence between the peak of adenosine at ZT12 and the maximal mitophagic flux is consistent with this model. It should be noted that while our genetic data establish that AdoR is required for BLT-induced mitophagy, they do not distinguish between AMPK acting downstream of AdoR, in parallel with it, or independently of the PINK1 axis. It would be valuable for future studies to incorporate direct AMPK activity measurements and genetic or pharmacological manipulations in order to explore this possibility and to define the hierarchical relationship between energy-sensing pathways and AdoR-mediated mitophagy.

Notwithstanding the advances that have been made, there are still important limitations and future directions to be addressed. While the use of *Drosophila* models enables rigorous causal analysis, there is a need for mammalian studies to clarify light–adenosine pathways, including retinal–hypothalamic signaling, astrocyte adenosine release, equilibrative nucleoside transporter activity, and cell type-specific adenosine receptor functions.

## 5. Conclusions

In summary, our study provides evidence that bright light exposure is associated with improved phenotypes in Tau-overexpressing *Drosophila*, accompanied by increased adenosine availability and changes in mitochondrial homeostasis and mitophagy-related readouts. Our data support a functional involvement of AdoR-associated signaling in multiple BLT-associated outcomes and highlight mitochondrial quality control as a candidate downstream process. This framework provides a foundation for future studies aimed at defining the causal signaling cascade and evaluating the translational relevance of light-based interventions.

## Figures and Tables

**Table 1:** Primary antibodies used in Western blot analysis.

Antibody	Dilution	Catalog No.	Source
*β*-actin	1:5000	A5316	Sigma-Aldrich, St. Louis, MI, USA
Tau	1:1000	PA5–27287	Invitrogen, Waltham, MA, USA
pTau	1:1000	44-758G	Invitrogen, Waltham, MA, USA
Atg8a	1:1000	ab109364	Abcam, CambridgeUK
PINK1	1:1000	A11435	Abclonal, Woburn, MA, USA
Parkin	1:1000	A0968	Abclonal, Woburn, MA, USA

## Data Availability

The raw data supporting the conclusions of this article will be made available by the authors on request.

## Supplementary Materials

The following supporting information can be downloaded at: https://www.mdpi.com/article/10.3390/biomedicines14071502/s1, Figure S1: Light exposure does not alter survival or locomotor performance in normal control flies; Figure S2: Light exposure extends survival across multiple neurodegenerative *Drosophila* models; Figure S3: Light exposure improves locomotor performance in multiple neurodegenerative *Drosophila* models; Figure S4: Time-course of adenosine levels in flies with or without BLT; Figure S5: Validation of *AdoR* knockdown efficiency by qRT-PCR; Figure S6: Comparison of mitophagy at ZT0 and ZT18 in Tau-overexpressing flies with or without BLT; Figure S7: Adenosine supplementation during the daytime improves short-term memory performance and attenuates neurodegeneration in Tau-overexpressing *Drosophila*; Figure S8: Adenosine supplementation enhances brain mitophagy in Tau-overexpressing *Drosophila*.

## References

[1] Ashleigh T., Swerdlow R.H., Beal M.F. (2023). The role of mitochondrial dysfunction in Alzheimer’s disease pathogenesis. Alzheimer’s Dement..

[2] Wang W., Zhao F., Ma X., Perry G., Zhu X. (2020). Mitochondria dysfunction in the pathogenesis of Alzheimer’s disease: Recent advances. Mol. Neurodegener..

[3] Abubakar M.B., Sanusi K.O., Ugusman A., Mohamed W., Kamal H., Ibrahim N.H., Khoo C.S., Kumar J. (2022). Alzheimer’s Disease: An Update and Insights Into Pathophysiology. Front. Aging Neurosci..

[4] Wittmann C.W., Wszolek M.F., Shulman J.M., Salvaterra P.M., Lewis J., Hutton M., Feany M.B. (2001). Tauopathy in *Drosophila*: Neurodegeneration without neurofibrillary tangles. Science.

[5] Zhou X., Du K., Mao T., Wang N., Zhang L., Tian Y., Liu T., Wang L., Wang X. (2024). BMAL1 upregulates STX17 levels to promote autophagosome-lysosome fusion in hippocampal neurons to ameliorate Alzheimer’s disease. iScience.

[6] Musiek E.S., Holtzman D.M. (2016). Mechanisms linking circadian clocks, sleep, and neurodegeneration. Science.

[7] Blackman J., Morrison H.D., Gabb V., Biswas B., Li H., Turner N., Jolly A., Trender W., Hampshire A., Whone A. (2023). Remote evaluation of sleep to enhance understanding of early dementia due to Alzheimer’s Disease (RESTED-AD): An observational cohort study protocol. BMC Geriatr..

[8] Liu C., Liou Y.M., Jou J. (2021). Pilot Study of the Effects of Bright Ambient Therapy on Dementia Symptoms and Cognitive Function. Front. Psychol..

[9] Riemersma-van Der Lek R.F., Swaab D.F., Twisk J., Hol E.M., Hoogendijk W.J., Van Someren E.J. (2008). Effect of bright light and melatonin on cognitive and noncognitive function in elderly residents of group care facilities: A randomized controlled trial. JAMA.

[10] Jagannath A., Varga N., Dallmann R., Rando G., Gosselin P., Ebrahimjee F., Taylor L., Mosneagu D., Stefaniak J., Walsh S. (2021). Adenosine integrates light and sleep signalling for the regulation of circadian timing in mice. Nat. Commun..

[11] Peng W., Liu X., Ma G., Wu Z., Wang Z., Fei X., Qin M., Wang L., Li Y., Zhang S. (2023). Adenosine-independent regulation of the sleep-wake cycle by astrocyte activity. Cell Discov..

[12] Lemmer B., Brühl T., Witte K., Pflug B., Köhler W., Touitou Y. (1994). Effects of bright light on circadian patterns of cyclic adenosine monophosphate, melatonin and cortisol in healthy subjects. Eur. J. Endocrinol..

[13] Reichert C.F., Deboer T., Landolt H. (2022). Adenosine, caffeine, and sleep-wake regulation: State of the science and perspectives. J. Sleep Res..

[14] Porkka-Heiskanen T., Alanko L., Kalinchuk A., Stenberg D. (2002). Adenosine and sleep. Sleep Med. Rev..

[15] Dolezelova E., Nothacker H.P., Civelli O., Bryant P.J., Zurovec M. (2007). A *Drosophila* adenosine receptor activates cAMP and calcium signaling. Insect Biochem. Mol. Biol..

[16] Liu Z., Wang F., Tang M., Zhao Y., Wang X. (2019). Amyloid beta and tau are involved in sleep disorder in Alzheimer’s disease by orexin A and adenosine A(1) receptor. Int. J. Mol. Med..

[17] Theparambil S.M., Kopach O., Braga A., Nizari S., Hosford P.S., Sagi-Kiss V., Hadjihambi A., Konstantinou C., Esteras N., Gutierrez Del Arroyo A. (2024). Adenosine signalling to astrocytes coordinates brain metabolism and function. Nature.

[18] Gnad T., Navarro G., Lahesmaa M., Reverte-Salisa L., Copperi F., Cordomi A., Naumann J., Hochhäuser A., Haufs-Brusberg S., Wenzel D. (2020). Adenosine/A2B Receptor Signaling Ameliorates the Effects of Aging and Counteracts Obesity. Cell Metab..

[19] Pickrell A.M., Youle R.J. (2015). The roles of PINK1, parkin, and mitochondrial fidelity in Parkinson’s disease. Neuron.

[20] Wang N., Yang J., Chen R., Liu Y., Liu S., Pan Y., Lei Q., Wang Y., He L., Song Y. (2023). Ginsenoside Rg1 ameliorates Alzheimer’s disease pathology via restoring mitophagy. J. Ginseng Res..

[21] Lazarou M., Sliter D.A., Kane L.A., Sarraf S.A., Wang C., Burman J.L., Sideris D.P., Fogel A.I., Youle R.J. (2015). The ubiquitin kinase PINK1 recruits autophagy receptors to induce mitophagy. Nature.

[22] Heo J.M., Ordureau A., Swarup S., Paulo J.A., Shen K., Sabatini D.M., Harper J.W. (2018). RAB7A phosphorylation by TBK1 promotes mitophagy via the PINK-PARKIN pathway. Sci. Adv..

[23] Kucerova L., Broz V., Fleischmannova J., Santruckova E., Sidorov R., Dolezal V., Zurovec M. (2012). Characterization of the *Drosophila* adenosine receptor: The effect of adenosine analogs on cAMP signaling in *Drosophila* cells and their utility for in vivo experiments. J. Neurochem..

[24] Qiao J., Yang S., Geng H., Yung W.H., Ke Y. (2022). Input-timing-dependent plasticity at incoming synapses of the mushroom body facilitates olfactory learning in *Drosophila*. Curr. Biol..

[25] Behnke J.A., Ye C., Moberg K.H., Zheng J.Q. (2021). A protocol to detect neurodegeneration in *Drosophila* melanogaster whole-brain mounts using advanced microscopy. STAR Protoc..

[26] Lee J.J., Sanchez-Martinez A., Martinez Zarate A., Benincá C., Mayor U., Clague M.J., Whitworth A.J. (2018). Basal mitophagy is widespread in *Drosophila* but minimally affected by loss of Pink1 or parkin. J. Cell Biol..

[27] Abhilash L., Shafer O.T. (2023). Parametric effects of light acting via multiple photoreceptors contribute to circadian entrainment in *Drosophila* melanogaster. Proc. Biol. Sci..

[28] Schmid E.T., Pyo J., Walker D.W. (2022). Neuronal induction of BNIP3-mediated mitophagy slows systemic aging in *Drosophila*. Nat. Aging.

[29] Jiménez-Loygorri J.I., Jiménez-García C., Viedma-Poyatos Á., Boya P. (2024). Fast and quantitative mitophagy assessment by flow cytometry using the mito-QC reporter. Front. Cell Dev. Biol..

[30] McWilliams T.G., Prescott A.R., Allen G.F., Tamjar J., Munson M.J., Thomson C., Muqit M.M., Ganley I.G. (2016). mito-QC illuminates mitophagy and mitochondrial architecture in vivo. J. Cell Biol..

[31] Rosignol I., Villarejo-Zori B., Teresak P., Sierra-Filardi E., Pereiro X., Rodríguez-Muela N., Vecino E., Vieira H.L.A., Bell K., Boya P. (2020). The mito-QC Reporter for Quantitative Mitophagy Assessment in Primary Retinal Ganglion Cells and Experimental Glaucoma Models. Int. J. Mol. Sci..

[32] Twig G., Elorza A., Molina A.J., Mohamed H., Wikstrom J.D., Walzer G., Stiles L., Haigh S.E., Katz S., Las G. (2008). Fission and selective fusion govern mitochondrial segregation and elimination by autophagy. EMBO J..

[33] Vincow E.S., Merrihew G., Thomas R.E., Shulman N.J., Beyer R.P., MacCoss M.J., Pallanck L.J. (2013). The PINK1-Parkin pathway promotes both mitophagy and selective respiratory chain turnover in vivo. Proc. Natl. Acad. Sci. USA.

[34] Clark I.E., Dodson M.W., Jiang C., Cao J.H., Huh J.R., Seol J.H., Yoo S.J., Hay B.A., Guo M. (2006). *Drosophila* pink1 is required for mitochondrial function and interacts genetically with parkin. Nature.

[35] Huang Y., Wan Z., Tang Y., Xu J., Laboret B., Nallamothu S., Yang C., Liu B., Lu R.O., Lu B. (2022). Pantothenate kinase 2 interacts with PINK1 to regulate mitochondrial quality control via acetyl-CoA metabolism. Nat. Commun..

[36] Quinn P.M., Moreira P.I., Ambrósio A.F., Alves C.H. (2020). PINK1/PARKIN signalling in neurodegeneration and neuroinflammation. Acta Neuropathol. Commun..

[37] Leng Y., Musiek E.S., Hu K., Cappuccio F.P., Yaffe K. (2019). Association between circadian rhythms and neurodegenerative diseases. Lancet Neurol..

[38] Yang H., Niu L., Tian L., Hu Y., Cheng C., Li S., Le W. (2025). Circadian rhythm disturbances in Alzheimer’s disease: Insights from plaque-free and plaque-burdened stages in APP(SWE)/PS1(dE9) mice. Alzheimer’s Res. Ther..

[39] Cremascoli R., Sparasci D., Giusti G., Cattaldo S., Prina E., Roveta F., Bruno F., Ghezzi C., Cerri S., Picascia M. (2021). Effects of Circadian Phase Tailored Light Therapy on Sleep, Mood, and Cognition in Alzheimer’s Disease: Preliminary Findings in a Pivotal Study. Front. Physiol..

[40] Lin F., Su Y., Weng Y., Lin X., Weng H., Cai G., Cai G. (2021). The effects of bright light therapy on depression and sleep disturbances in patients with Parkinson’s disease: A systematic review and meta-analysis of randomized controlled trials. Sleep Med..

[41] Zang L., Liu X., Li Y., Liu J., Lu Q., Zhang Y., Meng Q. (2023). The effect of light therapy on sleep disorders and psychobehavioral symptoms in patients with Alzheimer’s disease: A meta-analysis. PLoS ONE.

[42] Cornelissen T., Vilain S., Vints K., Gounko N., Verstreken P., Vandenberghe W. (2018). Deficiency of parkin and PINK1 impairs age-dependent mitophagy in *Drosophila*. eLife.

[43] Singh F., Ganley I.G. (2021). Parkinson’s disease and mitophagy: An emerging role for LRRK2. Biochem. Soc. Trans..

