# Supplementary material for: Light Exposure Ameliorates Tau-Induced Deficits via Adenosine Signaling and Mitochondrial Quality Control in Drosophila
Source: Biomedicines. 2026 Jul 2;14(7):1502. doi: 10.3390/biomedicines14071502 (PMC13405969; doi:10.3390/biomedicines14071502)
Supplement: Supplementary file 1 [file biomedicines-14-01502-s001.zip › biomedicines-4313099-supplementary.pdf]

Supplementary Information

Light exposure ameliorates Tau-induced deficits via adenosine signaling and mitochondrial quality control in *Drosophila*

Supplementary Figures

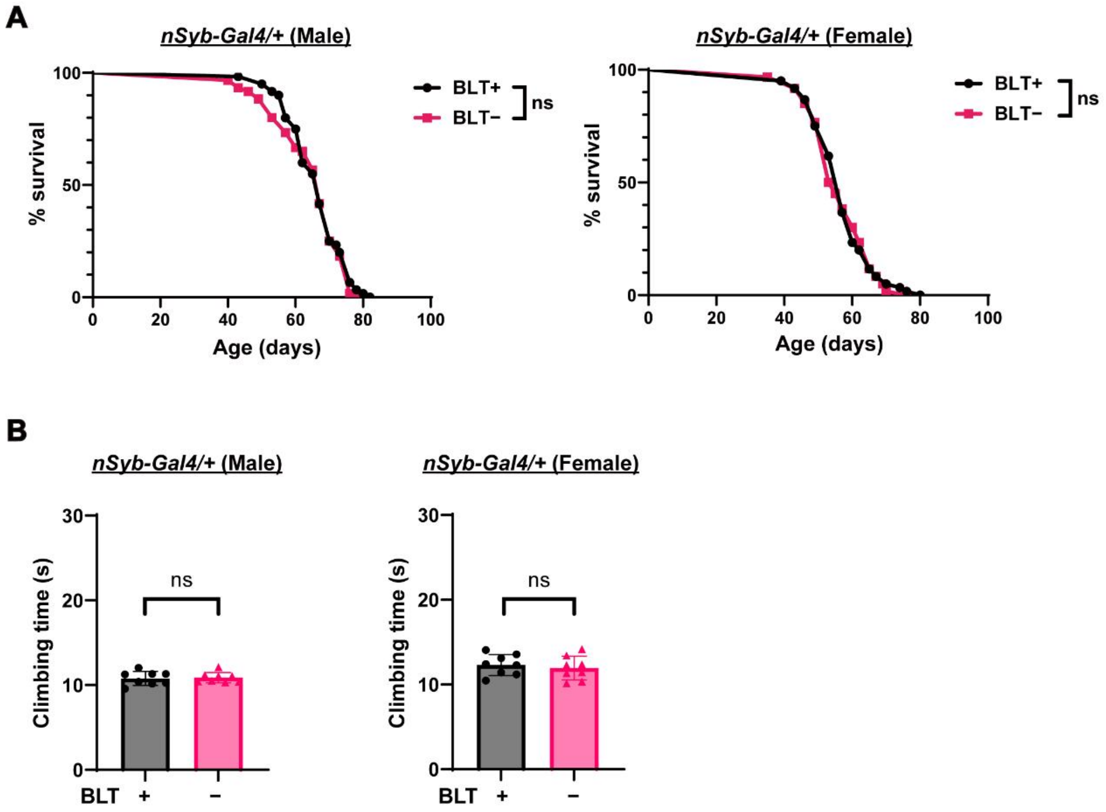

Supplementary Figure S1. Light exposure does not alter survival or locomotor performance in normal control flies. **(A)** Survival curves of normal control flies (*nSyb-Gal4*/+) under BLT+ and BLT− conditions. No significant differences were detected between BLT+ and BLT− in either sex. n = 60 biologically independent animals per group. ns, not significant; log-rank test. **(B)** Negative geotaxis (climbing) performance of normal control flies (*nSyb-Gal4*/+) under BLT+ or BLT− conditions. n = 8 replicates with 20 biologically independent animals per replicate. ns, not significant; unpaired *t* test. Data are presented as mean ± SD.

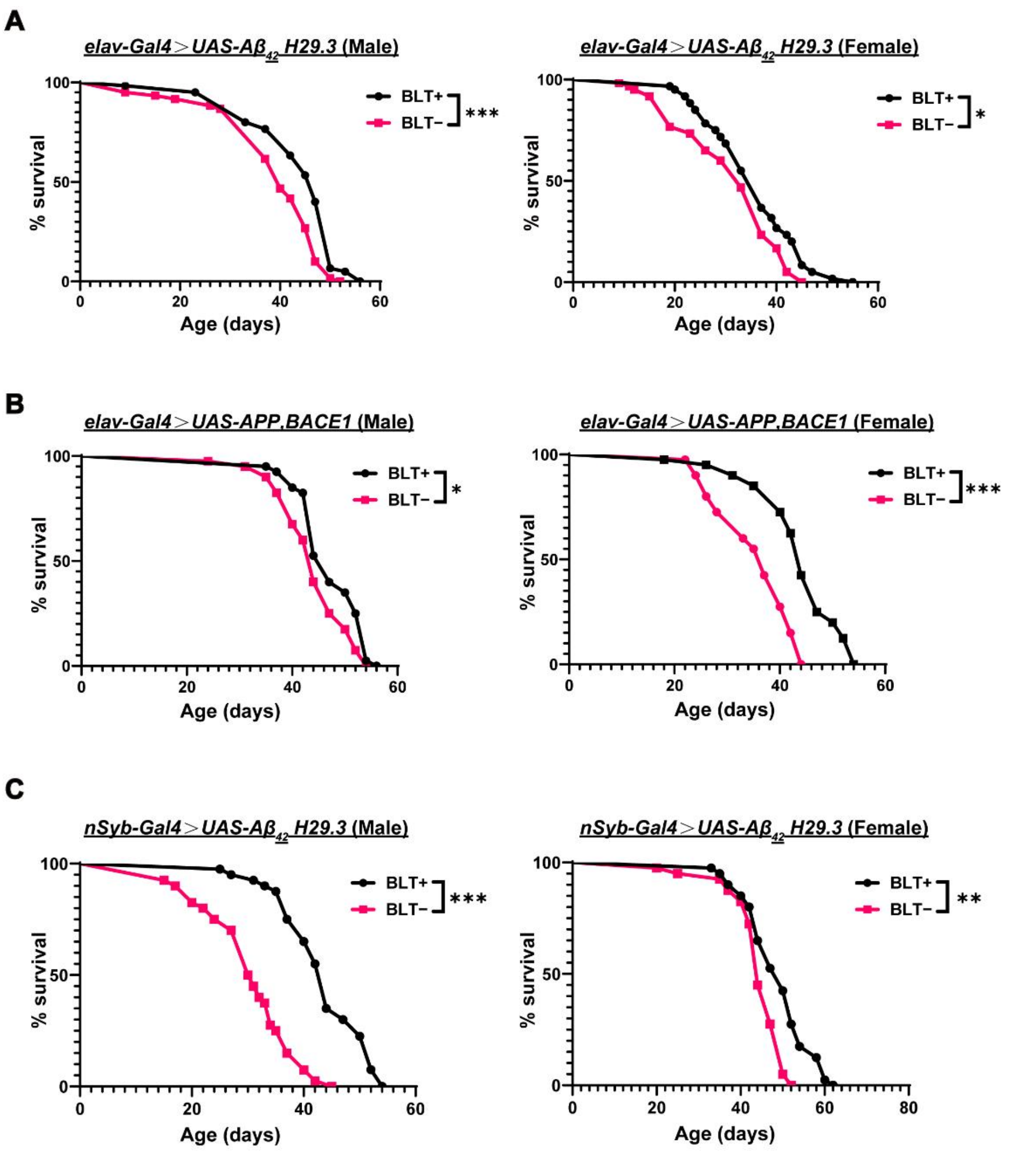

Supplementary Figure S2. Light exposure extends survival across multiple neurodegenerative *Drosophila* models. **(A-C)** Survival curves comparing BLT+ and BLT − conditions in different neurodegenerative models. For each genotype, left panels show males and right panels show females. n = 60 biologically independent animals per group. **p* < 0.05; ***p* < 0.01; log-rank test.

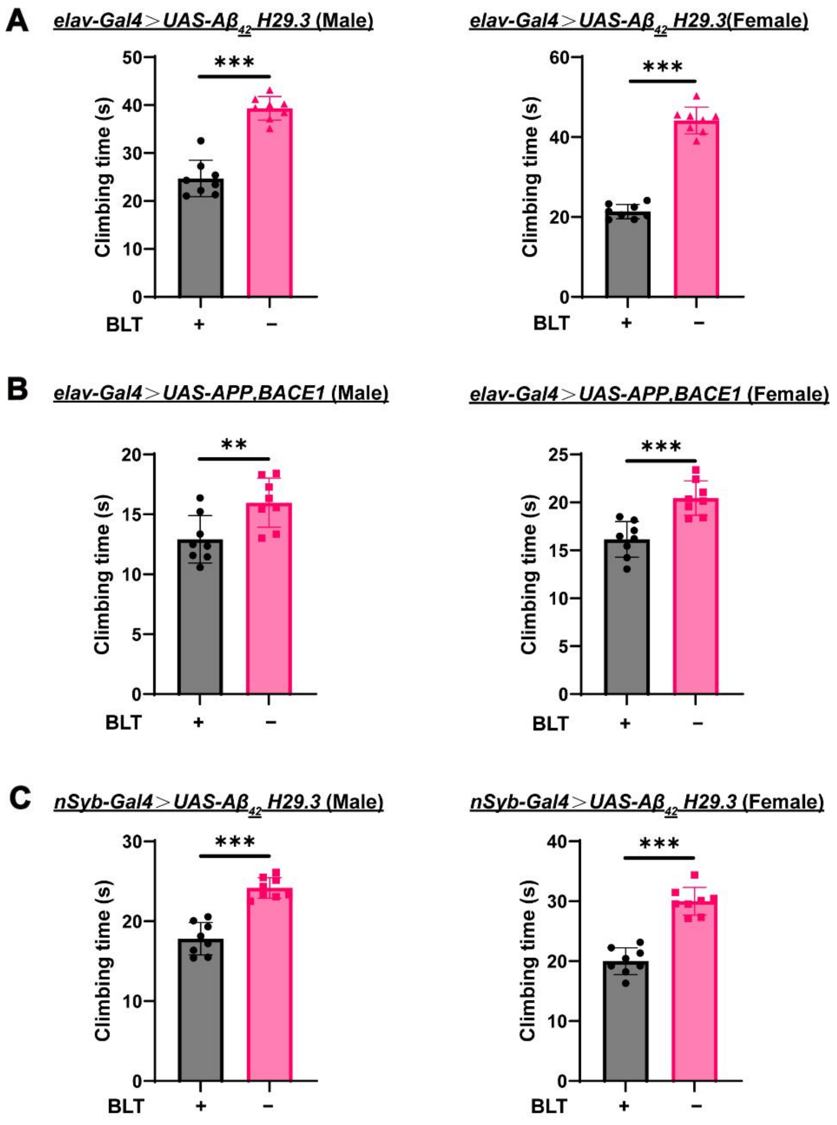

Supplementary Figure S3. Light exposure improves locomotor performance in multiple neurodegenerative *Drosophila* models. **(A-C)** Negative geotaxis (climbing) performance displayed as climbing time (seconds) under BLT+ and BLT− conditions. Lower values indicate faster climbing and improved locomotor function. Across genotypes and sexes, BLT+ groups show reduced climbing time relative to BLT−, indicating improved locomotor function. n = 8 replicates with 20 biologically independent animals per replicate. $^{**}p < 0.01$; $^{***}p < 0.001$; unpaired *t* test. Data are presented as mean ± SD.

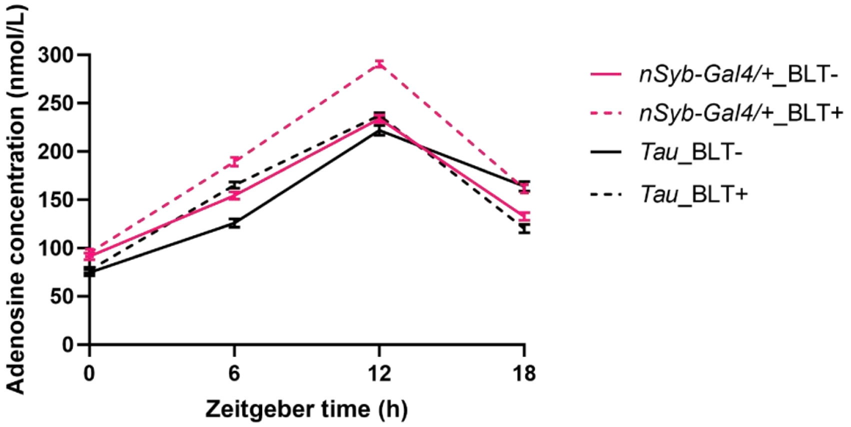

Supplementary Figure S4. Time-course of adenosine levels in flies with or without BLT. Adenosine concentrations were measured in heads of 30-day-old flies at four time points across the light-dark cycle (ZT0, ZT6, ZT12, and ZT18). n = 6 replicates per replicate. Data are presented as mean ± SD.

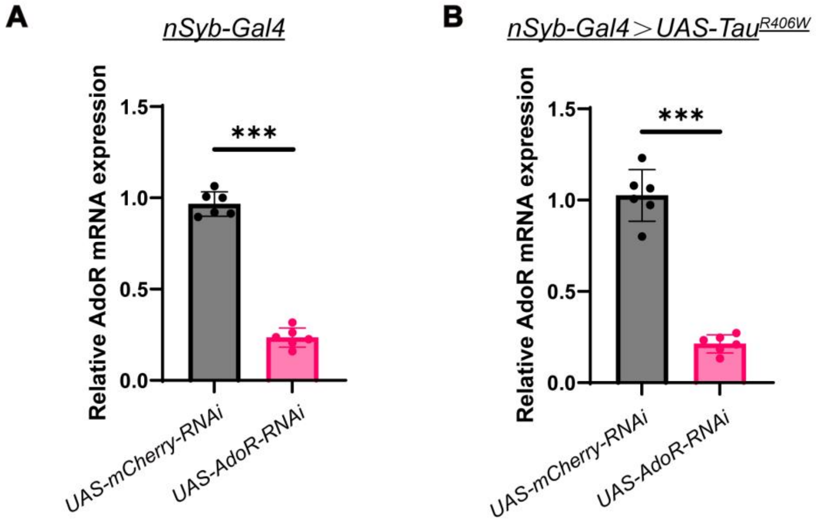

Supplementary Figure S5. Validation of *AdoR* knockdown efficiency by qRT-PCR. **(A)** Relative *AdoR* mRNA expression in heads of 30-day-old flies expressing either *mCherry-RNAi* (control) or *AdoR-RNAi* in the *nSyb-Gal4* background. **(B)** Relative *AdoR* mRNA expression in heads of 30-day-old flies expressing either *mCherry-RNAi* (control) or *AdoR-RNAi* in the *nSyb-Gal4* > *UAS-Tau*^{R406W} background. n = 6 replicates per replicate. ***$p < 0.001$; unpaired t test. Data are presented as mean ± SD.

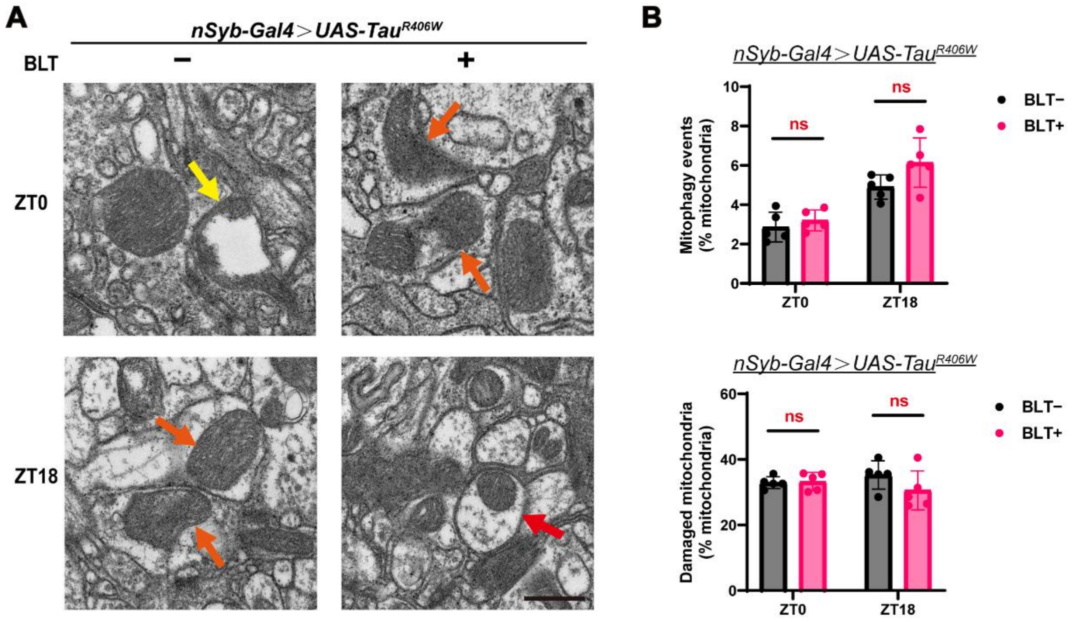

Supplementary Figure S6. Comparison of mitophagy at ZT0 and ZT18 in Tau-overexpressing flies with or without BLT. **(A)** Representative TEM images showing mitochondrial ultrastructure and mitophagic events at ZT0 (adenosine trough) and ZT18 (post-light clearance), with or without BLT. Orange arrows indicate moderately damaged mitochondria; yellow arrows indicate severely damaged mitochondria; red arrow indicates mitophagic events. Scale bar, 500 nm. **(B)** The percentage of mitochondria undergoing mitophagy-like events and the percentage of damaged mitochondria. ns, not significant. Data are shown as the mean ± SD.

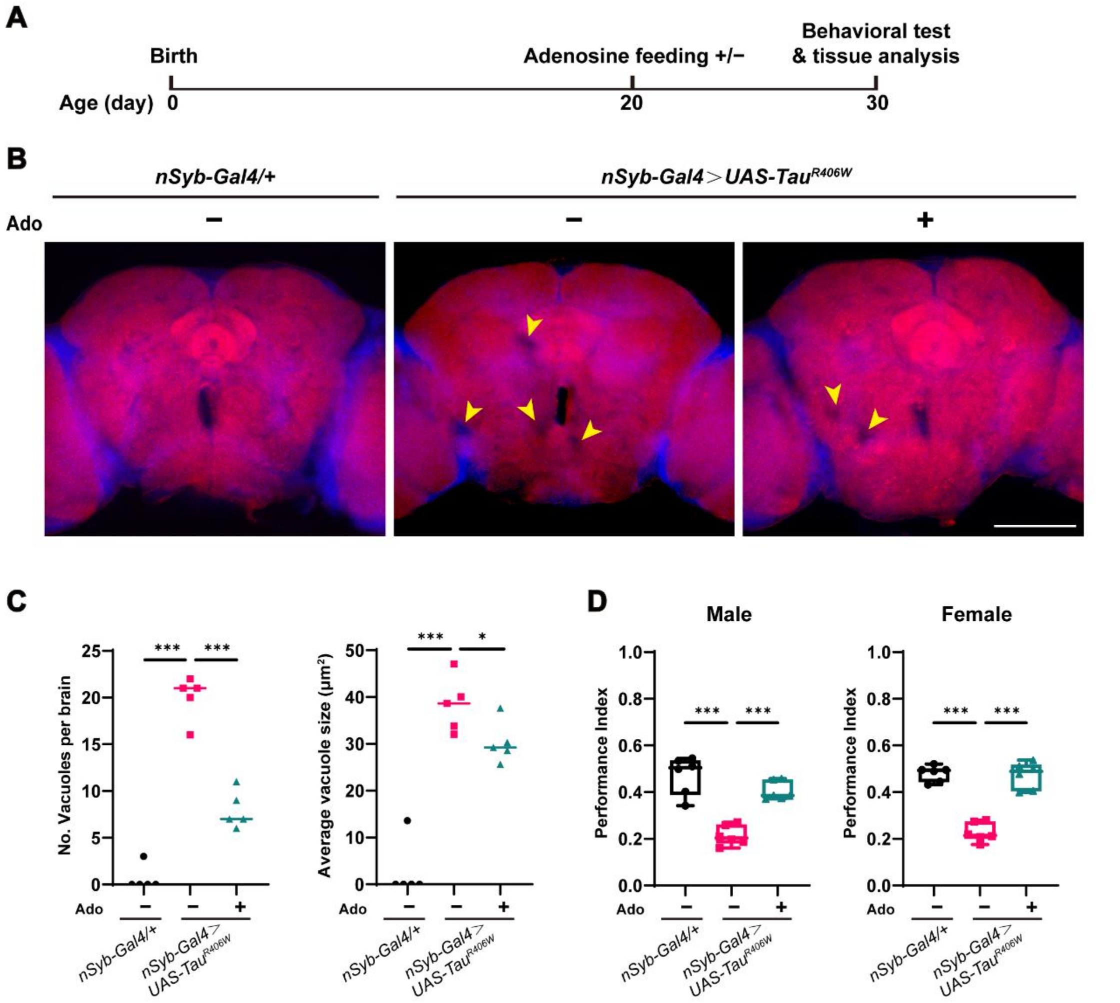

Supplementary Figure S7. Adenosine supplementation during the daytime improves short-term memory performance and attenuates neurodegeneration in Tau-overexpressing *Drosophila*. **(A)** Schematic diagram of the adenosine feeding protocol: adenosine was administered starting at day 20 post-eclosion for 10 consecutive days. **(B-C)** Representative fluorescence microscopy images of whole brains (B) and representative vacuoles indicated by yellow arrows. Scale bar, 100 μm. (C) Quantification showing that daytime adenosine administration significantly reduces the number and size of neurodegenerative vacuoles in Tau-overexpressing fly brains. n = 5 biologically independent animals. *p < 0.05; ***p < 0.001; unpaired t test. **(D)** Performance index in the T-maze olfactory learning assay. Adenosine-treated Tau-overexpressing flies exhibit significantly better performance index than untreated Tau-overexpressing flies. n = 6 biological replicates per condition. ***p < 0.001; unpaired t test. Data are presented as mean ± SD.

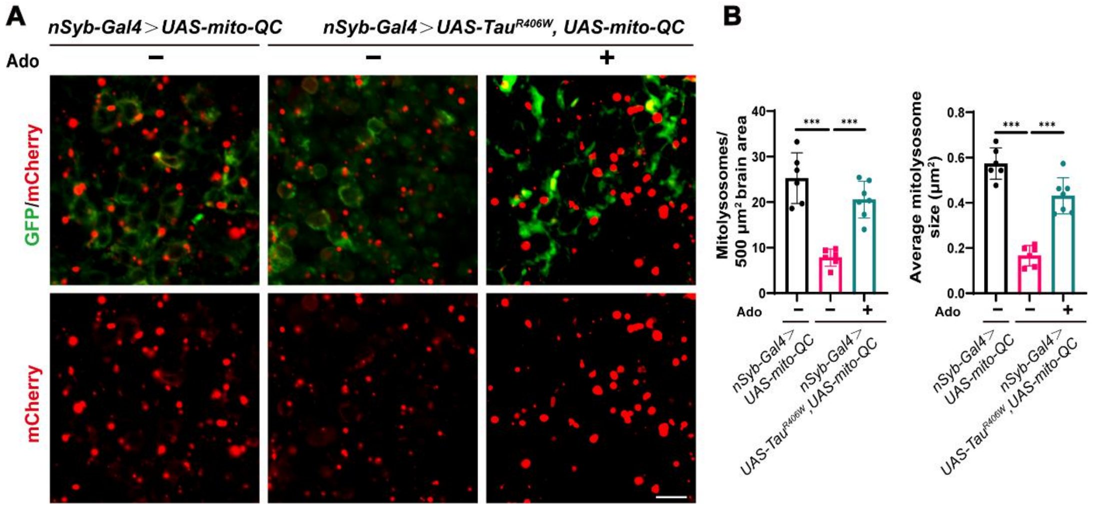

Supplementary Figure S8. Adenosine supplementation enhances brain mitophagy in Tau-overexpressing Drosophila. **(A)** Mito-QC imaging of brains from 30-day-old flies with or without adenosine feeding. Genotypes analyzed were *nSyb-Gal4* > *UAS-mito-QC* (control) and *nSyb-Gal4* > *UAS-Tau*^{R406W}, *UAS-mito-QC*. Images show merged GFP and mCherry channels along with mCherry-only puncta (mitolysosomes, where GFP has been quenched in the acidic lysosomal environment). Scale bar, 5 μm. (B) Quantification of mitolysosome area per 500 μm^2 and average size (μm^2) as shown in (A). n = 6 *UAS-mito-QC*, 6 *UAS-Tau*^{R406W}, *UAS-mito-QC* Ado−, and 7 *UAS-Tau*^{R406W}, *UAS-mito-QC* Ado+ biologically independent animals per condition, as indicated. ***$p < 0.001$; one-way ANOVA followed by Tukey's post hoc tests. Data are presented as mean ± SD.
